# A Randomized Placebo-Controlled Trial of Combination Therapy With Post-triple-antibiotic-therapy Fecal Microbiota Transplantation and Alginate for Ulcerative Colitis: Protocol

**DOI:** 10.3389/fmed.2022.779205

**Published:** 2022-02-22

**Authors:** Dai Ishikawa, Xiaochen Zhang, Kei Nomura, Natsumi Seki, Mayuko Haraikawa, Keiichi Haga, Tomoyoshi Shibuya, Yun-Gi Kim, Akihito Nagahara

**Affiliations:** ^1^Department of Gastroenterology, Juntendo University School of Medicine, Tokyo, Japan; ^2^Department of Intestinal Microbiota Therapy, Juntendo University School of Medicine, Tokyo, Japan; ^3^Research Center for Drug Discovery, Faculty of Pharmacy, Graduate School of Pharmaceutical Sciences, Keio University, Tokyo, Japan

**Keywords:** ulcerative colitis, alginate, fecal microbiota transplantation (FMT), double-blind randomized controlled trial, antibiotic-FMT, placebo-controlled clinical study

## Abstract

**Background:**

Fecal microbiota transplantation (FMT) has been widely performed for ulcerative colitis (UC) treatment at the clinical trial stage. Previous reports have used multiple FMT methods to enhance the colonization of healthy donor microbiota in the recipient's intestines. FMT following triple antibiotic therapy with amoxicillin, fosfomycin, and metronidazole (A-FMT) is not only effective but also requires only one FMT, which improves dysbiosis caused by reduced Bacteroidetes diversity in patients with UC. Alginate and its derivatives have the potential to induce the growth of intestinal bacteria including Bacteroides members and produce short-chain fatty acids (SCFAs), which are beneficial in regulating overactive autoimmunity. Our trial aims to investigate whether post-intervention with alginate, which can improve the intestinal environment, will enhance the therapeutic effect of A-FMT in UC and increase the long-term remission rate.

**Methods and Analysis:**

This trial is a double-blinded, randomized, placebo-controlled, parallel assignment trial. Patients with UC and fecal donation candidates will undergo strict screening before being involved in the trial. Eligible patients are randomly divided into two groups: one group will drink one bottle of alginate twice a day for 8 consecutive weeks after A-FMT, while the other group will take a placebo instead of the alginate drink. The primary endpoints are the changes in the Total Mayo Score at 8 weeks after study initiation and A-FMT from baseline. The secondary endpoint is the comparison of clinical features, microbiota, and metabolomic analysis before and after 8 weeks of study food intake. Changes at 6, 12, 18, and 24 months after A-FMT will be assessed. Finally, a subpopulation analysis of the relationship between patients and donors is an exploratory endpoint.

**Discussion:**

The FMT post-treatment used in this study is an oral alginate drink that is easily accepted by patients. If the regimen achieves the desired results, it can further improve the A-FMT regimen and provide evidence for clinical practice guidelines for UC.

**Clinical Trial Registration:**

https://jrct.niph.go.jp/latest-detail/jRCTs031200103, identifier: jRCTs031200103.

## Introduction

Although advances in ulcerative colitis (UC) drug treatment regimens have been made, 20–25% of patients choose a surgical intervention due to resistance or intolerance to drug treatment, which can cause pain and inconvenience to patients (1). To date, the pathogenesis of UC is not completely understood, and is only known to be the result of autoimmune processes and genetic and environmental factors; however, patients with UC commonly show decreased diversity and richness in the intestinal microbiota, resulting in dysbiosis (2, 3). Dysbiosis is thought to contribute to the development of aberrant immunological responses in inflammatory bowel disease (IBD) (4).

Fecal microbiota transplantation (FMT) is a minimally invasive therapeutic approach that involves transplanting intestinal microbiota from healthy donors to restore the normal intestinal microbiota functions in patients with disease-related dysbiosis. The implementation of FMT to treat UC is rapidly attracting the attention of the public (5, 6). Randomized clinical trials have assessed the use of multiple FMT operations for enhancing the colonization of the intestinal microbiota from healthy donors (7–10). The clinical remission rate of patients receiving FMT reached an average of 42.1% (11). However, the methods outlined in the above reports are difficult to manage and implement in actual clinical practice.

FMT following triple antibiotic therapy with amoxicillin, fosfomycin, and metronidazole (A-FMT), a method used by our team, is simple to operate because it only requires one FMT *via* colonoscopy followed by administration of antibiotics for 2 weeks. The combination antibiotic therapy consisting of amoxicillin, fosfomycin, and metronidazole (AFM) was modified for amoxicillin, tetracycline and metronidazole (ATM) therapy to reduce the adverse events caused by tetracycline. The efficacy of these combination antibiotic therapies (AFM, ATM) in treating patients with UC was previously reported (12, 13). We employed AFM as antibiotic pre-treatment to enhance reprogramming of the host intestinal microbiota by increasing the donor microbe colonization (14) and to achieve synergistic effects with FMT. We previously reported that A-FMT contributed to the recovery of the Bacteroidetes composition, which is associated with clinical responses and UC severity (15). It has also been observed that Bacteroidetes species components in clinical responders treated with A-FMT remarkably resembled those of their donors (16). These results indicate that A-FMT can effectively transplant the Bacteroidetes cells which is lost as UC activity progress (17) in fecal specimens from the donor to the intestinal environment of the recipient, which is parallel to the clinical improvement of UC. Furthermore, the high average clinical remission rate (43.8%) was observed in patients at four weeks after administering A-FMT comparing to previous reports with conventional FMT methods (42.1%) (15, 18). Although A-FMT exhibited obvious advantages compared to AFM monotherapy in our long-term clinical study, the remission rate gradually declined to 18.2% within 24 months (18). Wei et al. reported that intervention with pectin, a soluble dietary fiber extracted from apples, after FMT can preserve the diversity of the intestinal microbiota of patients with UC, providing results similar to those of donors (19). We speculate that FMT combined with post-interventions to maintain the diversity of the intestinal microbiota may improve the efficacy of FMT in UC treatment.

Alginic acid is a soluble dietary fiber polysaccharide that is widely distributed in the cell walls of brown algae in the form of alginate (20). Previous reports have found that alginate oligosaccharides have various activities, such as anti-inflammatory activity (21), antifungal activity (22) and immunomodulatory activity (23). Besides, alginate not only has a protective function on mucous membranes of the upper gastrointestinal tract (24), but also ameliorated the symptoms of experimental colitis and inflammatory responses (25). Mirshafiey et al. have also verified that alginate can be used as a potential treatment option for UC in both acute and chronic phase by rat models (26, 27). Furthermore, alginate and its derivatives can improve the growth of intestinal bacteria and can be fermented by human gut bacteria to produce short-chain fatty acids (SCFAs), which are beneficial to the intestine (28–30). A meta-analysis showed that clinical improvements in IBD are associated with the abundance of fecal microbiota and the enrichment of SCFA-producing anaerobes (31). Therefore, the aims of our study are to determine whether post-intervention with alginate can increase the diversity of microbiota to enhance the therapeutic effect of A-FMT in UC and delay the loss of microbiota diversity to improve the long-term remission rate. To our knowledge, this is the first randomized controlled study of the relationship between alginate and UC therapy.

## Methods and Analysis

### Study Design and Patients

This double-blinded randomized, placebo-controlled, parallel assignment trial will be conducted at Juntendo University Hospital (Tokyo, Japan). The study will be conducted until December 31, 2024 and is expected to involve 60 patients with UC. Diagnosis of UC will be established based on standard clinical, endoscopic, and histological findings (32) and both hospitalized patients and outpatients will be included. To be eligible, patients must be diagnosed with active UC and meet the requirements of obtaining a Total Mayo Score of 3–10 (7, 9, 10) and a Sum Endoscopic Mayo Score of 2 or above. All patients will be over 20 years old and competent enough to provide informed consent. There are no restrictions on gender.

To ensure the safety of patients, rigorous screening of patients will be performed. If the patient has serious illness, pregnant women, or has received local therapy, etc. will not be included in our experiment. The detailed exclusion criteria for this study are listed in Table 1.

**Table 1:** Exclusion criteria for patients with UC.

Informed consent not provided
Infectious enterocolitis
Receiving local therapy
Serious disease, such as liver disease, kidney disease, heart disease, or other serious complications
Autoimmune disease
Pregnant women and all cases with the possibility of pregnancy
Allergic diseases
Antibiotic therapy in the past 3 months
Any other cases judged inappropriate by the responsible researcher

*UC, Ulcerative colitis*.

### Donors

To understand the health status of the donors and reduce additional costs, we will conduct a preliminary medical inquiry and physical examination of the donor candidates. As dysbiosis is correlated with neuropsychiatric disorders including depression and autism (gut-brain interaction) (33, 34), the Hamilton Depression Scale (HAMD) and Neuropsychiatric Inventory (NPI) will be used to assess psychiatric symptoms (Table 2A). There are no restrictions on gender. In addition, to minimize the risk of infection transmission, the donor candidates who have passed the preliminary screening will undergo fecal and blood tests. Donor candidates who test positive for any of the items in Tables 2B, 2C will be excluded. Candidates who pass the above rigorous screening will become eligible donors. Finally, eligible donors will confirm whether they have diarrhea, fever, or other uncomfortable symptoms when submitting stool, as well as whether any cohabitants have diarrhea symptoms. The donor candidates recommended by the patients themselves will also need to pass all the screening processes mentioned above. Approximately 150–200 g of fresh stool provided by the eligible donors will be dissolved in 500 ml of sterile normal saline. The sample will be processed to filter out crude fiber, and 100% glycerol will be added to make the final glycerol concentration 10%. The sample will then be divided into 200-ml bottles (Corning Co., Ltd., NY, USA). This procedure will be performed under an anaerobic environment by replacing air with nitrogen gas in a glove box (SANPLATEC Co., Ltd., Osaka, Japan). The fecal suspension will be cryopreserved at −60°C in advance.

**Table 2A:** Donor exclusion criteria.

**Medical interview (exclusions)**
Age < 18 or >70
BMI < 18 or >25 or metabolic syndrome
Informed consent not provided
International travel to area with high risk of traveler's diarrhea in the last 6 months
High-risk sex (unprotected sex outside of a monogamous relationship in last 3 months, or men who have sex with men, sex for drugs or money)
Tattoo, body piercing, or acupuncture in the last 6 months
Needle stick accident in the last 6 months
Household members with active gastrointestinal infection
History of vaccination with a live attenuated virus in the last 3 months
Incarceration or a history of incarceration
**Medical history (exclusions)**
History of major gastrointestinal surgery
Family history of colorectal carcinoma
Active medical illness or symptoms
Antimicrobials (antibiotics, antivirals, antifungals), probiotics, or PPIs in the last 3 months
Taking any medications
Acute diarrhea in the last 3 months
Irritable bowel syndrome, chronic constipation, Chronic diarrhea Other intrinsic gastrointestinal illness: Inflammatory bowel disease, Colonic polyps, Colon cancer
Autoimmune disease
Atopic disease (including atopic dermatitis)
Chronic fatigue syndrome
**Psychiatric symptoms (exclusion)**
Any psychiatric disorder assessed by HAMD or NPI

*BMI, body mass index; PPIs, proton pump inhibitors; HAMD, Hamilton Depression Scale; NPI, Neuropsychiatric inventor*.

**Table 2B:** Donor screening criteria: blood test.

**Infections**
Hepatitis A virus IgM, Hepatitis B virus surface antigen/antibody, Hepatitis B virus core antibody, Hepatitis C virus antibody, Hepatitis E virus IgA
HIV type 1 and type 2 antibody and antigen
Human T-cell lymphotropic virus-1 antibody
Syphilis (RPR/TP)
Parasite-specific antibody screening test
Epstein Barr virus IgM
Cytomegalovirus antigen pp65 * RT-PCR
Tuberculosis (IFN-γ)
COVID-19 antigen
**Health condition**
Complete blood count
Electrolytes (sodium, potassium, chlorine)
Renal function tests (blood urea nitrogen, creatinine)
Liver function tests (AST, ALT, ALP, γ-GT)
Albumin
C-reactive protein

*Ig, immunoglobulin; HIV, human immunodeficiency virus; IFN, interferon; RPR, rapid plasma regain; TP, treponema pallidum; RT-PCR, reverse transcription-polymerase chain reaction; AST, aspartate transaminase; ALT, alanine transaminase; ALP, alkaline phosphatase; γ-GT, gamma-glutamyltransferase; SARS-CoV-2, severe acute respiratory syndrome corona virus 2*.

**Table 2C:** Donor screening criteria: fecal test.

Fecal occult blood testing
Parasites, ova, cysts
Cryptosporidium
Giardia
Norovirus
Rotavirus
General bacterial culture for common enteric pathogens
*Salmonella*
*Shigella*
*Yersinia*
*Campylobacter*
*Escherichia coli*
Diarrheagenic *Escherichia coli*
Enterohemorrhagic *Escherichia coli*
*Escherichia coli* verotoxin
*Clostridium difficile* toxin/ *Clostridium difficile*-specific GDH

*GDH, glutamate dehydrogenase*.

### Interventional Methods

First, eligible patients with UC will receive a combination antibiotic regimen called AFM comprising oral amoxicillin (1,500 mg/day), fosfomycin (3,000 mg/day), and metronidazole (750 mg/day). AFM will be administered to patients for 2 weeks until 2 days before the FMT (15). The fecal suspension (200 ml) will be thawed at 37°C using a water bath shaker on the day of the FMT operation. After bowel lavage using a standard polyethylene glycol (PEG) solution (Moviprep; EA Pharma, Tokyo, Japan), the patients will undergo total colonoscopy. A total of 200 mL of fecal suspension will be transferred to the patient's cecum. After the procedure, patients will receive scopolamine (10 mg) to slow intestinal transit and maintain a right lateral position. If the patient's drug dose for UC treatment has been stable for at least 12 weeks before the pre-registration, the patient will be permitted to continue ongoing treatment. However, changes in the dosage of drugs or initiation of new treatments will not be allowed during the intervention. After the A-FMT regimen described above, the patients will be randomized into two groups for a double-blind FMT post-treatment. One group will consume one bottle of alginate drink twice a day for 8 consecutive weeks, while the other group will take a placebo. The alginate drink is a mixed tea drink provided by Kaigen Pharma Co., Ltd. (Osaka, Japan) containing 4 g of sodium alginate per bottle (150 g) and has been proven to be safe (https://www.h-food.or.jp/dbadm/media/231cec6ad1478e2d429f733d93a7b272.pdf). The placebo uses the same tea drink with the sodium alginate removed and does not have a different appearance or taste. Patients can adjust their medication during post-observation after the study food intervention. To compare the effects of treatment, all patients will undergo symptom checking and colonoscopy and have blood and fecal samples tested at the times indicated in Table 3. Colonoscopy founding at screening, A-FMT and 8 weeks after study food intake should be taken video and judged by a third-part expert to confirm the scope and severity of UC lesions. Biopsy of inflammatory sites will be used to evaluate the histological score. Blood samples will be used to test routine blood biochemistry, and plasma components will be separated by centrifugal force and cryopreserved at −60°C. Fecal samples will be collected to determine the occult blood condition and calprotectin levels and analyze the intestinal microbiota and metabolites.

**Table 3:** Protocol for sequential therapy of A-FMT with alginate drink or placebo.

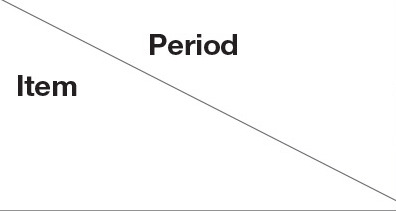	**Screening stage**		**Duration of administration**				**Post-observation**
	**Pre-registration (within 2 months)**	**Screening (within a month)**	**AFM**	**A-FMT**	**Alginate drink or Placebo**		**6, 12, 18, and 24 months after**
			**2 weeks**		**4 weeks after**	**8 weeks after**	
Informed consent	•						
Check of patient background	•						
Administration of study food					•	•	
Check of symptoms	•	•		•	•	•	•
Endoscopy		•		•		•	
Blood samples		•		•		•	•
Fecal samples		•		•		•	•

*A-FMT, FMT following triple antibiotic therapy AFM (amoxicillin, fosfomycin, and metronidazole)*.

### Outcomes

The primary endpoint is to compare the changes in the Total Mayo Score at 8 weeks after study initiation and A-FMT from baseline between alginate intake group and placebo intake group. The secondary endpoints are the comparison of the following items at 8 weeks after study initiation and A-FMT from baseline:

- The change in the Sum Endoscopic Mayo Score.
- Analysis of intestinal microbiota by 16S rRNA gene amplicon sequencing and whole-genome sequencing.
- Metabolomic analysis: In addition to SCFAs in stool samples, the amount of lipids, free fatty acids, bile acids, and other clinical markers will also be measured by liquid chromatography tandem mass spectrometry (35).

We will also do same analysis as (1), (2), and (3) at 6, 12, 18, and 24 months after 8 weeks post-study initiation.

Subpopulation analysis of the relationship between patients and donors is considered an exploratory endpoint.

Clinical features will be judged by the Total Mayo Score, and the mucosal appearance of the Mayo Score will be evaluated by endoscopy. To evaluate the overall status of the intestine, endoscopic findings will be evaluated using the Sum Endoscopic Mayo Score, which refers to the sum of the scores of the seven segments (appendicular region, cecum, ascending colon, transverse colon, descending colon, sigmoid colon, and rectum). Intestinal microbial analysis will assess changes in composition and diversity. Metabolomic analysis will evaluate the metabolites of microbiota and elucidate the mechanism of action of alginate in the treatment of UC.

### Adverse Events

Any adverse event caused by antibiotic (12), FMT therapy, study food intake, blood sampling, and endoscopy should be recorded in detail, including symptoms, time, duration, and follow-up until complete resolution or termination of treatment. If there is any serious adverse event that may lead to death, life-threatening, or intolerability, the study will be terminated and treatment will begin immediately.

### Progress and Estimated Study Period

This trial began on August 25, 2020. The first study participant was enrolled on September 28, 2020. At present, 33 eligible patients have been included in our study on December 8, 2021. We summarized the baseline characteristics of the 33 patients (Table 4). If the number of participants continues to increase at this pace, this trial is expected to end in October 2022.

**Table 4:** The baseline characteristics of the study participants.

**Items**	**All (*n* = 33)**
Age	40.5 ± 12.0
Sex (M/F)	22/11
Duration of disease (years)	7.3 ± 5.4
**Disease location**	
Proctitis	4
Left-sided colitis	14
Extensive colitis	15
Total Mayo score	7.12 ± 1.9
Mild: 3–5	8
Moderate: 6–10	25
Sum endoscopic mayo score	6.5 ± 4.2
**Ongoing treatment**	
5-ASA	30
Corticosteroid	2
Azathioprine	4
Vedolizumab	1
Anti-TNF	3

*5-ASA, 5-aminosalicylic acid; Anti-TNF, anti-tumor necrosis factor*.

### Sample Size Estimation

In our previous clinical trials (15), ~40 eligible patients with UC were collected in 1 year. Therefore, conservative estimates predict that 60 patients can be collected over 3 years. Eligible patients with UC will be randomized and assigned to two groups with a ratio of 1:1 in a double-blinded fashion. The estimated difference between each group in the Total Mayo Score as the primary endpoint from baseline is −2, and the standard deviation of each group is estimated to be ~2.5. If a two-sample *t*-test is used to test the difference between groups, a power analysis predicts that statistical significance with a power of ~86.1% can be reached.

### Statistical Analysis

Relationships between Total Mayo Scores and Sum Endoscopic Mayo Scores will be assessed using a *t*-test. Furthermore, the correlation between the relative abundance of Bacteroidetes species using a Pearson's correlation coefficient will be evaluated. Differences will be considered significant at *P* < 0.05.

### Randomization

Eligible patients will be randomized at a ratio of 1:1 using an Interactive Web Response System (REDCap: Research Electronic Data CAPture). The simple randomization is carried out by the staff in charge of the clinical research and trial Center of the Juntendo University Hospital to ensure concealment of allocation. The medical staff, assistants and patients of our study team did not know the condition of random allocation until the experiment was completely finished.

## Discussion

FMT is a well-established treatment regimen for recurrent *Clostridium difficile* infection (CDI). Clinical practice guidelines clearly state that FMT can be used as a treatment option for CDI (36, 37). However, using FMT for UC treatment remains in the clinical trial stage. Since 2014, we have been committed to studying how to enhance the therapeutic effect and operational feasibility of FMT on UC in clinical trials. The proposed A-FMT method has a significant effect on the treatment of UC in our previous studies. We hope to combine acceptable and convenient post-treatment methods based on A-FMT to further enhance the effect and prolong the remission period, including alginate supplements.

Alginate has the potential to increase the relative abundance of some beneficial Bacteroidetes members, resulting in an improved intestinal environment (38–40). Therefore, we hypothesize that the diversity of microbiota and the proportion of Bacteroidetes in the group treated with A-FMT combined with alginate will be higher than that in the placebo group. An increased number of Bacteroidetes species can suppress the inflammatory response through zwitterionic capsular polysaccharides, which are bacterial products that modulate T cells to secrete the anti-inflammatory interleukin-10 (41). It has also been reported that alginate can be fermented by specific bacteria in feces, such as *Bacteroides ovatus, Bacteroides xylanisolvens*, and *Bacteroides theaiotaomicron* (30, 42), which can induce colonic regulatory T cells and promote anti-inflammatory effects (28, 43). Therefore, we believe that oral supplementation of alginate with exogenous sources can contribute to the intestinal anti-inflammatory response. Furthermore, we expect that the group treated with A-FMT combined with alginate will demonstrate better results in terms of clinical features and microbial analysis, and will retain its advantage over the placebo arm after 2 years' follow-up. The metabolites produced by microbiota can shape the colonic environment through a variety of activities, such as participating in signaling, immune system modulation, and antibiotic activity (44–46). However, it is not fully known how specific microbes and the small molecules they modulate interact to cause or control the inflammatory response. Therefore, in our study, a wide range of metabolomic analyses other than SCFAs will be included in the study.

Although this clinical study is limited to patients in single center, our method can also be applied to patients in other regions, then provide more evidence for the treatment of patients with UC.

## Ethics Statement

The study protocol was approved by the Ethics Committee of the Juntendo Institutional Review Board, Juntendo University School of Medicine, and the Clinical Study Committee of Juntendo University Hospital (Approval Number J20-011). The patients/participants provided their written informed consent to participate in this study.

## Author Contributions

DI and Y-GK: conceptualization. DI: methodology, validation, funding acquisition, writing—review and editing, and project administration. DI and KN: formal analysis, data curation, and visualization. KH and TS: investigation. XZ and NS: writing—original draft preparation. AN: supervision. MH, KH, and TS: resources. All authors have read and agreed to the published version of the manuscript.

## Funding

This research was funded by the joint research course with Kirin Holdings Co., Ltd. The funder was not involved in the study design, collection, analysis, interpretation of data, the writing of this article or the decision to submit it for publication.

## Conflict of Interest

Kaigen Pharma Co., Ltd (Osaka, Japan) provided the alginate drink and placebo for this clinical study. The authors declare that the research was conducted in the absence of any commercial or financial relationships that could be construed as a potential conflict of interest. The reviewer TO declared a shared affiliation with several of the authors, DI, XZ, KN, MH, KH, TS, and AN, to the handling editor at time of review.

## Publisher's Note

All claims expressed in this article are solely those of the authors and do not necessarily represent those of their affiliated organizations, or those of the publisher, the editors and the reviewers. Any product that may be evaluated in this article, or claim that may be made by its manufacturer, is not guaranteed or endorsed by the publisher.

## Abbreviations

UC: ulcerative colitis
IBD: inflammatory bowel disease
FMT: fecal microbiota transplantation
A-FMT, FMT following triple antibiotic therapy AFM (amoxicillin, fosfomycin: and metronidazole)
FCSA: short-chain fatty acids
CDI: *Clostridium difficile* infection
HAMD: Hamilton Depression Scale
NPI: Neuropsychiatric Inventory
PEG: polyethylene glycol.
